# The effect of aging on the biological and immunological characteristics of periodontal ligament stem cells

**DOI:** 10.1186/s13287-020-01846-w

**Published:** 2020-07-29

**Authors:** Xiaoyu Li, Bowen Zhang, Hong Wang, Xiaolu Zhao, Zijie Zhang, Gang Ding, Fulan Wei

**Affiliations:** 1 Department of Orthodontics, School and Hospital of Stomatology, Cheeloo College of Medicine, Shandong University & Shandong Key Laboratory of Oral Tissue Regeneration & Shandong Engineering Laboratory for Dental Materials and Oral Tissue Regeneration, No.44-1 Wenhua Road West, Jinan, 250012 Shandong China; 2 Department of Stomatology, Yidu Central Hospital, Weifang Medical University, Qingzhou, Shandong China

**Keywords:** Periodontal ligament stem cells, Peripheral blood mononuclear cells, Osteogenic differentiation, Immunosuppression, Aging, Tissue engineering

## Abstract

**Background:**

Periodontal ligament stem cells (PDLSCs) have many applications in the field of cytotherapy, tissue engineering, and regenerative medicine. However, the effect of age on the biological and immunological characteristics of PDLSCs remains unclear.

**Methods:**

In this study, we compared PDLSCs isolated from young and adult individuals. PDLSC proliferation was analyzed by Cell Counting Kit-8 (CCK-8) and 5-ethynyl-2′-deoxyuridine (EdU) staining, and apoptosis level was detected by Annexin V-PE/7-ADD staining. PDLSC osteogenic/adipogenic/chondrogenic differentiation potentials were assessed by alkaline phosphatase (ALP), Alizarin Red, Oil Red O, Alcian Blue staining, and related quantitative analysis. PDLSC immunosuppressive capacity was determined by EdU and Annexin V-PE/7-ADD staining. To explore its underlying mechanism, microarray, quantitative reverse transcriptase-polymerase chain reaction (qRT-PCR), and western blot analyses were performed to detect differentially expressed genes and proteins in PDLSCs.

**Results:**

Our results demonstrated that with aging, the proliferation and osteogenic/adipogenic/chondrogenic differentiation potential of PDLSCs decreased, whereas apoptosis of PDLSCs increased. Moreover, the immunosuppressive ability of PDLSCs decreased with aging. Compared with PDLSCs from young subjects, analysis of mRNA expression revealed an upregulation of *CCND3* and *RC3H2*, and a downregulation of *Runx2*, *ALP*, *COL1A1*, *PPARγ2*, *CXCL12*, *FKBP1A*, *FKBP1B*, *NCSTN*, *P2RX7*, *PPP3CB*, *RIPK2*, *SLC11A1*, and *TP53* in those from adult individuals. Furthermore, protein expression levels of Runx2, ALP, COL1A1, and PPARγ2 in the adult group were decreased, whereas that of CCND3 increased.

**Conclusions:**

Taken together, aging influences the biological and immunological characteristics of PDLSCs, and thus, it is more appropriate to utilize PDLSCs from young individuals for tissue regeneration, post-aging treatment, and allotransplantation.

## Introduction

Mesenchymal stem cells (MSCs) are pluripotent non-hematopoietic progenitor cells that have the capability of self-renewal and multi-directional differentiation and thus play a key role in tissue regeneration [1]. Furthermore, its low immunogenicity and strong immunoregulation have allowed the use of MSCs in cytotherapy for allotransplantation of stem cells and the treatment of severe autoimmune diseases [2, 3]. As a kind of MSCs, periodontal ligament stem cells (PDLSCs) are not only easy to be harvested, but also have strong self-renewal and multilineage differentiation potential, which can provide a reliable cell origin for clinical applications such as periodontal regeneration therapy [4, 5]. In addition, PDLSCs can inhibit the proliferation of allogeneic peripheral blood mononuclear cells (PBMCs), thus providing a good foundation for the application of PDLSCs in allogeneic transplantation [6]. However, previous studies have shown that age-related changes in the stem cell population and function can significantly affect the eventual clinical regeneration of cells [7].

According to the research data, we know that the regeneration potential of MSCs is an age-related degeneration [8]. Previous studies have shown that aging can reduce the proliferation and osteogenic differentiation ability of bone marrow mesenchymal stem cells (BMSCs) and increase their adipogenic differentiation ability; this may be related to bone senescence, in which BMSCs differentiate into fat cells rather than bone cells with age [9, 10]. However, other studies have shown that the adipogenic differentiation ability of BMSCs decreases with age [11]. At present, the differences in the adipogenic differentiation ability of BMSCs remain unclear. Wu et al. have earlier shown that age is an important factor that leads to weakened biological properties of PDLSCs [12]. With increasing age, the proliferation and osteogenic/adipogenic differentiation ability of PDLSCs decrease [13]. PDLSCs have many characteristics that are similar to BMSCs; thus, it is essential to determine whether donor age influences the biological characteristics of PDLSCs.

Previous studies have shown that the immunosuppressive potential of MSCs derived from bone marrow, adipose, and other sources is negatively influenced by aging [14, 15]. In our previous studies, we showed that PDLSCs not only have the ability to reconstruct the periodontal tissue and bone defects [16, 17], but also have low immunogenicity and strong immune regulation ability [18]. Several studies have focused on the immunomodulatory potential of PDLSCs in animals and humans [19, 20]. In vitro, PDLSCs inhibit mixed lymphocyte responses or non-specific mitogen-induced T lymphocyte proliferation in a dose-dependent manner [21]. In vivo, Ding et al. [20] and Park et al. [22] found that periodontal stem cells are beneficial for the treatment of a variety of inflammatory and immune diseases. Previous studies have demonstrated that the anti-inflammatory and immunosuppressive effects of MSCs depend on several different pathways, including direct cell contact and secretion of soluble factors [23, 24], such as transforming growth factor-β (TGF-β), prostaglandin E2 (PGE2), hepatocyte growth factor (HGF), indoleamine 2, 3-dioxygenase (IDO), nitric oxide (NO), and interleukin-6 (IL-6). PDLSCs can inhibit the proliferation of allogeneic T lymphocytes, which is the result of the combined effect of direct cell contact and secretion of soluble factors [18, 21]. However, the effect of age on the immunological properties of PDLSCs and its underlying mechanism has not been studied to date.

Therefore, we investigated the effect of age on the biological and immunological characteristics of PDLSCs and screened factors which differed with age. Our experimental data provide additional evidence for the effect of age on the characteristics of PDLSCs. In addition, these results provide an important theoretical basis for later cell therapy and tissue regeneration applications.

## Materials and methods

### Isolation and culture of PDLSCs

Our dental samples consisted of intact third molars extracted from 20 systemically healthy donors who were young (YPDLSCs range 19–20 years) and 21 systemically healthy donors who were adult (APDLSCs range 35–50 years) who provided their informed consent. All the procedures were approved by the Research Ethics Committee of Shandong University (no. G201401601). The periodontal ligament tissue in the middle third of the root surface was scraped and seeded into flasks, then maintained in the complete culture medium containing α-MEM (HyClone) and 10% fetal bovine serum (FBS, Gibco) at 37 °C in 5% CO_2_. The medium was refreshed every 2 to 3 days. In the present study, APDLSCs and YPDLSCs between passages 3–4 (P3–4) were used to avoid cell behavioral changes that were related to prolonged culture such as senescence in vitro. For each experiment, the same passages APDLSCs and YPDLSCs were used. In each experiment, the young group had three independent donors and the adult group had three independent donors.

### Flow cytometric analysis

The cell surface markers of APDLSCs and YPDLSCs were analyzed by flow cytometry (BD Biosciences) according to the manufacturer’s instructions. Briefly, after washing with PBS containing 3% FBS, the cells were incubated with monoclonal antibodies against human CD105, STRO-1, CD146, CD31, CD45, HLA-І, HLA-II DR, CD80, and CD86 in the dark at 4 °C for 1 h; cells without pretreatment by any antibody were used as blank control. Subsequently, the cells were washed with PBS and analyzed by flow cytometry.

### Cell proliferation assay

Cell proliferation was quantitatively detected using a Cell Counting Kit-8 (CCK-8) (Dojindo Laboratories) and 5-ethynyl-2′-deoxyuridine (EdU, Ribobio) incorporation assay according to the manufacturer’s instructions. Briefly, in the CCK-8 assay, the APDLSCs and YPDLSCs were seeded into 96-well culture plates separately (2 × 10^3^ cells/well) and incubated for 1, 2, 3, 4, 5, 6, and 7 days. At each prescribed time point, the medium was replaced by fresh α-MEM supplemented with 10% CCK-8 solution and then incubated at 37 °C in the dark for 2 h before measuring the absorbance at a wavelength of 450 nm using a microplate reader (SPECTROstar Nano; BMG Labtech). For the EdU labeling assay, the APDLSCs and YPDLSCs were seeded into 12-well culture plates separately (6 × 10^4^ cells/well) and incubated for 72 h. Then, 50 μM EdU labeling medium was added to each well at 37 °C in the dark for 2 h, and the cells were fixed and then stained by Apollo®567 and Hoechst33342. The wells were then visualized with a fluorescence microscope (Olympus), and images were captured. The proliferation rate of the cells was examined based on the proportion of EdU-positive nuclei (red) to blue fluorescent nuclei by counting three microscopic fields randomly for each well.

### Cell apoptosis assay

The percentage of apoptotic cells was detected by Annexin V-PE/7-ADD staining kit (CWBIO). APDLSCs and YPDLSCs were seeded into six-well culture plates separately (1 × 10^5^ cells/well) for apoptosis detection. Upon reaching 80–90% confluency, the cells were treated with a binding buffer after washing with cold PBS. Finally, the cells were harvested, resuspended in 100 μL of binding buffer containing 5 μL Annexin V-PE and 10 μL 7-ADD for 15 min in darkness at room temperature. The percentage of apoptotic cells was then detected by flow cytometry.

### Multilineage differentiation

For the osteogenic differentiation assays, APDLSCs and YPDLSCs were cultured separately with osteogenic inductive medium (complete medium supplemented with 50 mg/L vitamin C, 10 mmol/L sodium β-glycerophosphate, and 10 mmol/L dexamethasone); the medium was refreshed every 3 days. After 7 days, the PDLSCs were stained with ALP. The level of ALP activity in the cells was measured using an ALP activity assay kit (Nanjing Jiancheng Bioengineering Institute) according to the manufacturer’s instructions, and the absorbance was measured by a microplate reader at a wavelength of 520 nm. After 21 days, the mineralized nodules were stained with Alizarin Red. The area of the Alizarin Red stain was measured by a microplate reader at a wavelength of 560 nm after solubilizing in 10% cetylpyridinium chloride (CPC, Sigma-Aldrich) for 30 min at the room temperature.

For the adipogenic differentiation assays, APDLSCs and YPDLSCs were cultured separately with adipogenic inductive medium (complete medium supplemented with 1 μmol/L dexamethasone, 200 μmol/L indomethacin, 10 mg/L insulin, and 500 μmol/L IBMX); the medium was refreshed every 3 days. After 14 days, the lipid droplets were stained by Oil Red O. The area of the Oil Red O stain was measured by a microplate reader at a wavelength of 510 nm after solubilizing in isopropanol (Sigma-Aldrich) for 30 min at the room temperature.

For the chondrogenic differentiation assays, APDLSCs and YPDLSCs were cultured separately with chondrogenic inductive medium (StemPro® Chondrogenesis Differentiation Kit, Gibco); the medium was refreshed every 3 days. After 14 days, the synthesis of proteoglycans by chondrocytes was stained by Alcian Blue. The area of the Alcian Blue stain was measured by a microplate reader at a wavelength of 600 nm after solubilizing in guanidine hydrochloride solution (Sigma-Aldrich) overnight at 4 °C.

### Culture and identification of PBMCs

Peripheral blood was obtained from systemically healthy donors with ages between 16 and 19 years with their informed consent. Two milliliters of fresh heparinized peripheral blood was diluted with phosphate-buffered saline (PBS; HyClone); then, it was carefully overlayered onto 5 mL of Ficoll (1.077 g/mL) and centrifuged at 2000 rpm for 30 min. The lymphocyte layer was separated and washed with PBS, and the precipitated cells were resuspended in RPMI-1640 medium (Gibco) containing 10% FBS, 100 U/mL penicillin, and 100 μg/mL streptomycin.

### Immune assay

#### Effects of PDLSCs in different age groups on the proliferation of PBMCs

In vitro lymphocyte proliferation experiment can be used as an in vitro model to simulate in vivo cellular immune regulation. Non-specific stimulant PHA is often used to activate PBMCs, so as to indirectly observe the proliferation changes of PBMCs after recognizing specific antigens. APDLSCs and YPDLSCs were added to six-well plates separately (5 × 10^4^ cells/well) and cultured at 37 °C in 5% CO_2_ for 2 h to allow adherence to the substrate. Then, allogeneic PBMCs were added (5 × 10^5^ cells/well) and stimulated with 5 μg/mL phytohemagglutinin (PHA; Sigma-Aldrich), cultured in RPMI-1640 medium containing 10% fetal bovine serum, 100 U/mL penicillin, and 100 μg/mL streptomycin. After 5 days of co-culture, EdU (RiboBio) was added to the plates 8 h before harvesting the cells. Then, PBMC proliferation was analyzed by flow cytometry.

#### Delayed admission of PDLSCs from different age groups on proliferation of PBMCs

The rationale of delaying the addition of PDLSCs was to study whether PDLSCs in different age groups could inhibit the proliferation of activated PBMCs. PBMCs were stimulated (5 × 10^5^ cells/well) with 5 μg/mL PHA and cultured in RPMI-1640 medium containing 10% fetal bovine serum, 100 U/mL penicillin, and 100 μg/mL streptomycin for 2 days at 37 °C in 5% CO_2_. After 2 days, APDLSCs and YPDLSCs were added to the above reaction system separately (5 × 10^4^ cells/well) and co-cultured for 3 days. EdU was added to the plates 8 h before harvesting the cells. Then, PBMC proliferation was analyzed by flow cytometry.

#### Effect of PDLSCs from different age groups on two-way mixed lymphocyte response

In the field of transplantation, mixed lymphocyte response (MLR) has long been the basis for evaluating the compatibility of donor and recipient MHC II-like antigens and the clinical selection of donor. MLR is a method to detect donor-receptor compatibility, which can simulate the allogenic activation reaction in vitro. APDLSCs and YPDLSCs were added to six-well plates separately (5 × 10^4^ cells/well) and cultured at 37 °C in 5% CO_2_ for 2 h to allow adherence to the substrate. Then, PBMCs from two individuals were added (5 × 10^5^ cells/well) and stimulated with 5 μg/mL PHA and cultured in RPMI-1640 medium containing 10% fetal bovine serum, 100 U/mL penicillin, and 100 μg/mL streptomycin. After 5 days of co-culture, EdU was added to the plates 8 h before harvesting the cells. Then, PBMC proliferation was analyzed by flow cytometry.

#### Restimulation of PBMCs

The rationale of reactivating PBMCs was to compare whether PBMCs inhibited by PDLSCs of different age groups can be reactivated and whether their proliferation ability is different after reactivation. APDLSCs and YPDLSCs were added to six-well plates separately (5 × 10^4^ cells/well) and cultured at 37 °C in 5% CO_2_ for 2 h to allow adherence to the substrate. Then, allogeneic PBMCs were added (5 × 10^5^ cells/well) and stimulated with 5 μg/mL PHA and cultured in RPMI-1640 medium containing 10% fetal bovine serum, 100 U/mL penicillin, and 100 μg/mL streptomycin. After 5 days of co-culture, the supernatant was centrifuged and resuspended in culture medium. Then, 5 μg/mL PHA was added, and the culture was incubated for 2 days to reactivate the PBMCs. EdU was added to the plates 8 h before harvesting the cells, and PBMC proliferation was analyzed by flow cytometry.

#### Transwell culture of PBMCs

The rationale of Transwell culture system was to verify whether PDLSCs in different age groups have different ability to inhibit PBMCs proliferation when PDLSCs are not in direct contact with PBMCs. APDLSCs and YPDLSCs were added to 12-well plates separately (5 × 10^4^ cells/well) and cultured at 37 °C in 5% CO_2_ for 2 h to allow adherence to the substrate. Transwell chambers were used to separate the PBMCs from the PDLSCs. Allogeneic PBMCs were seeded (5 × 10^5^ cells/well) with PHA in the bottom chamber. After 5 days of co-culture, EdU was added to the plates 8 h before harvesting the cells, and PBMC proliferation was analyzed by flow cytometry.

#### Determination of the percentage of apoptotic PBMCs

The rationale of determining the percentage of PBMC apoptosis is to detect whether PDLSCs in different age groups exert different immunosuppressive effects by inducing apoptosis of PBMCs. APDLSCs and YPDLSCs were added into six-well plates separately (5 × 10^4^ cells/well) and cultured at 37 °C in 5% CO_2_ for 2 h to allow adherence to the substrate. Then, allogeneic PBMCs were added (5 × 10^5^ cells/well) and stimulated with 5 μg/mL PHA and cultured in RPMI-1640 medium containing 10% fetal bovine serum, 100 U/mL penicillin, and 100 μg/mL streptomycin. The percentage of apoptotic PBMCs after 5 days of co-culture was evaluated using the Annexin V-PE/7-ADD staining kit (CWBIO) according to the manufacturer’s instructions.

### Microarray assay

Microarray analysis was performed by LC-Bio Technology Co., Ltd. Total RNA was derived respectively from APDLSCs and YPDLSCs using TRIzol reagent (Invitrogen) according to the manufacturer’s protocols. The Affinity Script-RT kit (Agilent Technologies) and Promoter Primer were used to reverse transcribe RNA into the first strand of cDNA, and then the Anti-sense Promoter was used to generate the second strand of cDNA, and T7 RNA polymerase was added to generate cRNA by amplification of the second strand of cDNA. Then, Cyanine-3-CTP (Cy3) was used for labeling, after which RNeasy Kit (QIAGEN) was used for purification. Finally, hybridization was performed at 65 °C for 17 h, and the original image was scanned by Agilent Scanner G5761A (Agilent Technologies) after elution. Feature Extraction software (version 12.0.3.1, Agilent Technologies) was used to process the original image and extract the original data, and Genespring software (version 14.8, Agilent Technologies) was used for quantile standardization and subsequent processing. Genes with a *p* value < 0.05 or fold change > 2 were identified as differentially expressed. Subsequently, GO functional annotation analysis of the differentially expressed genes was performed.

### qRT-PCR analysis

Total RNA was isolated from PDLSCs of individuals of different age groups using the RNAios Plus reagent (Takara) and was reverse transcribed to cDNA using the PrimeScript TM RT reagent Kit with gDNA Eraser (Takara). In the present study, mRNA quantification was done for 18 identified genes, including *Runx2*, *ALP*, *COL1A1*, *PPARγ2*, *CCND3*, *RC3H2*, *PPP3CB*, *CXCL12*, *FKBP1A*, *FKBP1B*, *NCSTN*, *P2RX7*, *RIPK2*, *SLC11A1*, *TP53*, *TNFSF14*, *RC3H1*, and *TNFRSF4*. Then, qRT-PCR reactions were performed in a 10-μL reaction volume with the TB Green PCR Core Kit (Takara). GAPDH was used as an internal control to quantify and normalize the results. The information on the primers is shown in Table 1.

**Table 1:** Gene primers

Gene names	Sense primers	Antisense primers
Runx2	GTTTCACCTTGACCATAACCGT	GGGACACCTACTCTCATACTGG
ALP	TCCATCTGTAAAGGGCGGTAAT	AATACCAGCTACGCTGCATCAAG
COL1A1	GCTGATGATGCCAATGTGGTT	CCAGTCAGAGTGGCACATCTTG
PPARγ2	CTCCTATTGACCCAGAAAGC	GTAGAGCTGAGTCTTCTCAG
CCND3	CTGTTCCCCCTTCACAAAGC	CTAGCCACCGAAATGCAGAC
RC3H2	GCGTTGAGGATTTGGCACTC	GCATGGCTCTTACACGACCT
PPP3CB	ATGAGAGAATGCCACCTCGG	CGAGATGTGAGAGTCCCTGG
CXCL12	GGACTTTCCGCTAGACCCAC	GTCCTCATGGTTAAGGCCCC
FKBP1A	TCCAGATTATGCCTATGGTGCC	ATCGAAGACGAGAGTGGCAT
FKBP1B	TGCTCCAAAATGGGAAGAAGT	GCTGCACCCTCTTCAAAACC
NCSTN	GAAAGGGAGAACCAGCCGAA	GGAGTAAACACCAAACCCATCA
P2RX7	GGAGCCAAAGCCGACATTAAA	TGTGAAGTCCATCGCAGGTC
RIPK2	CAGCCTTTTGAAGATGTCACCA	ATCATACGTGCTCGGTGAGG
SLC11A1	ACTTGTCGGGCCTCAATGAT	ACCTTGTTCAGCAGGCCATT
TP53	AGTCACAGCACATGACGGAG	GCCAGACCATCGCTATCTGA
TNFSF14	GCTGTTATGGGAGACTCAGC	GCTGCACCTTGGAGTAGATG
RC3H1	GGTGCAAAGATTGGAGCCAC	CGGTGCAATTCGTAAGCCTG
TNFRSF4	GACCAACTGCACCTTGGCTG	TGTCCTCACAGATTGCGTCC
GAPDH	TCATGGGTGTGAACCATGAGAA	GGCATGGACTGTGGTCATGAG

*Runx2* runt-related transcription factor 2; *ALP* alkaline phosphatase; *COL1A1* collagen type I alpha 1; *PPARγ2* peroxisome proliferator activated receptor-gamma 2; *CCND3* cyclin D3; *RC3H2* ring finger and CCCH-type domains 2; *PPP3CB* protein phosphatase 3, catalytic subunit, beta isozyme; *CXCL12* chemokine (C-X-C motif) ligand 12; *FKBP1A* FK506 binding protein 1A; *FKBP1B* FK506 binding protein 1B; *NCSTN* nicastrin; *P2RX7* purinergic receptor P2X, ligand gated ion channel, 7; *RIPK2* receptor-interacting serine-threonine kinase 2; *SLC11A1* solute carrier family 11 (proton-coupled divalent metal ion transporter), member 1; *TP53* tumor protein p53; *TNFSF14* tumor necrosis factor (ligand) superfamily, member 14; *RC3H1* ring finger and CCCH-type domains 1; *TNFRSF4* tumor necrosis factor receptor superfamily, member 4; *GAPDH* glyceraldehyde 3-phosphate dehydrogenase

### Western blot analysis

APDLSCs and YPDLSCs were washed three times with ice-cold PBS and lysed using RIPA reagent containing 1% PMSF and 1% phosphatase inhibitor cocktail. After centrifugation at 12,000 rpm for 5 min, total protein concentrations were measured using a BCA Protein Assay Kit (Solarbio). Then, proteins were separated by sodium dodecyl sulfate-polyacrylamide gel electrophoresis (SDS-PAGE) according to molecular weight and transferred onto polyvinylidene difluoride (PVDF) membranes (Millipore). The membranes were blocked with 5% milk for 2 h and then incubated with primary antibodies overnight at 4 °C. Next, the membranes were washed three times with Tris-buffered saline solution with Tween-20 (TBS-T) and incubated with secondary antibodies at room temperature for 1 h. The protein bands were then developed with the use of the Enhanced Chemiluminescence (ECL) Substrate Kit (Millipore), and the densitometry of each band was conducted using ImageJ (National Institutes of Health). The following primary antibodies were used: Runx2 (1:1000, CST), ALP (1:30,000, Abcam), COL1A1 (1:1000, CST), PPARγ2 (1:500, Abcam), CCND3 (1:2000, CST), and GAPDH (1:10,000, Abcam).

### Statistical analysis

In each experiment, the young group had three independent samples from 3 different donors and the adult group had three independent samples from 3 different donors. All the results were presented as mean ± SD of three independent experiments. For a comparison of three or more groups, we performed one-way analysis of variance (ANOVA) test; and then, for a comparison of only two groups, we used Student’s *t* test. The statistical analyses were conducted by Prism (GraphPad Prism v7.02), and the values of *P* < 0.05 were considered to be statistically significant.

## Results

### Characterization of PDLSCs from different age groups

**Fig. 1:**
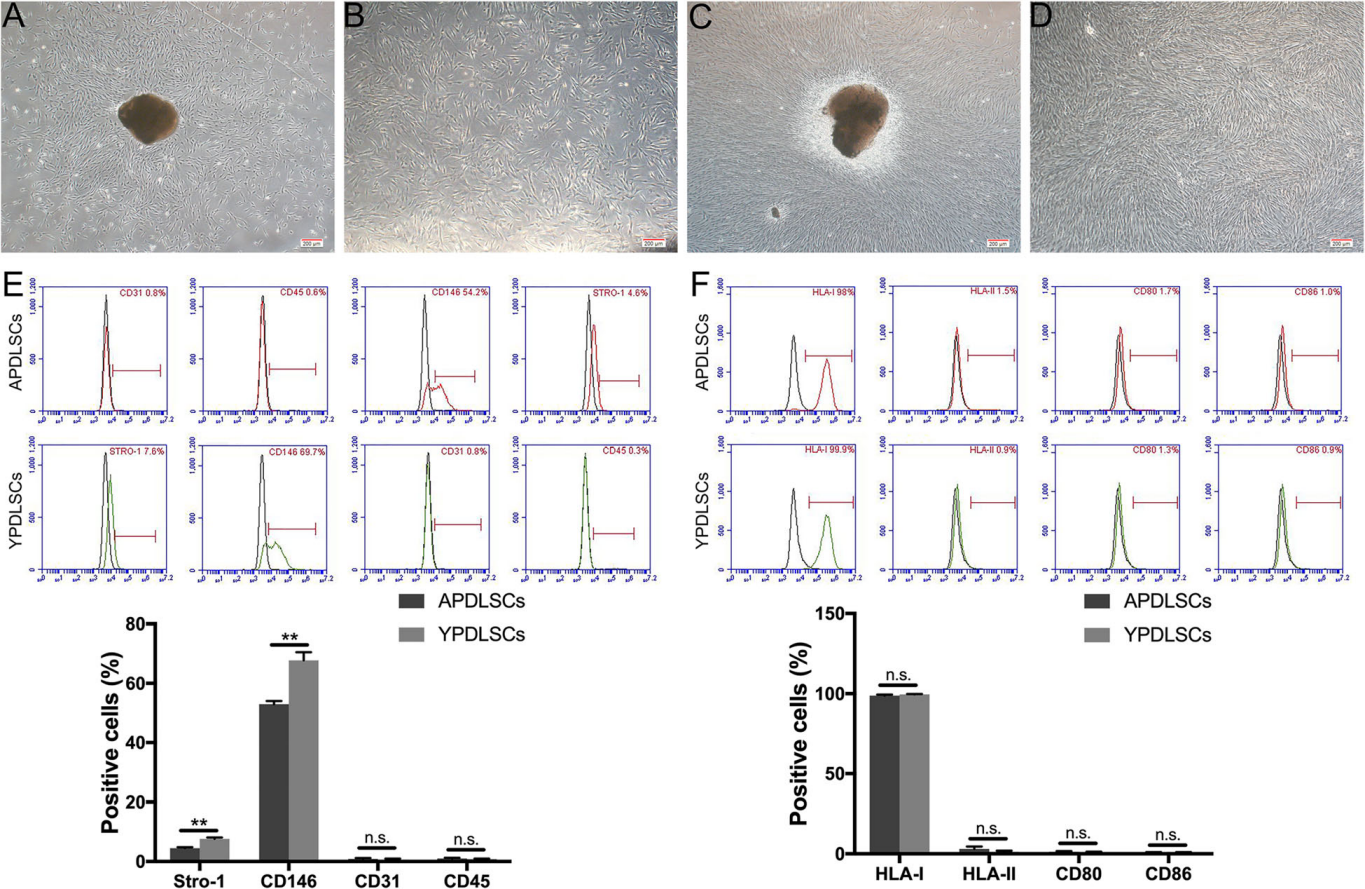
Characterization of PDLSCs from different age groups. **a** APDLSCs were cultured for 14 days (scale bar 200 μm). **b** APDLSCs of the third generation (P3) (scale bar 200 μm). **c** YPDLSCs were cultured for 14 days (scale bar 200 μm). **d** YPDLSCs of the third generation (P3) (scale bar 200 μm). **e** Both APDLSCs and YPDLSCs expressed STRO-1 and CD146 positively and expressed CD45 and CD31 negatively. However, the expression levels of STRO-1 and CD146 in APDLSCs were significantly lower than YPDLSCs. **f** Both APDLSCs and YPDLSCs expressed HLA-I positively and expressed HLA-II DR, CD80, and CD86 negatively. The young group had three independent donors and the adult group had three independent donors. Data are presented as mean ± SD of three independent experiments (**p < 0.01, N.S., no significance)

The obtained cells were arranged into APDLSCs and YPDLSCs according to the age of the donors. Evaluation using an inverted microscope revealed that the APDLSCs had irregular morphology and more bifurcated cell edges, whereas the YPDLSCs had long and spindle-shaped, with abundant cytoplasm and clear cell edges (Fig. 1a–d). The expression of cell-surface molecule CD105, an MSC maker was positive in both groups; there was no significant difference between APDLSCs and YPDLSCs (Additional file 1: Fig. S1). Positive expression of MSC-specific surface MSCs STRO-1 and CD146 was observed in the APDLSCs and YPDLSCs; the rate of positivity in APDLSCs was much lower than that in YPDLSCs. Meanwhile, the expression of leukocyte-specific molecule CD45 and platelet endothelial cell-specific molecule CD31 was negative in both groups; there was no significant difference between APDLSCs and YPDLSCs (Fig. 1e). In addition, both APDLSCs and YPDLSCs expressed HLA-I, but not HLA-II DR and costimulatory molecules CD80 and CD86; there was no significant difference between the two groups (Fig. 1f).

### Proliferation and apoptosis of PDLSCs from different age groups

**Fig. 2:**
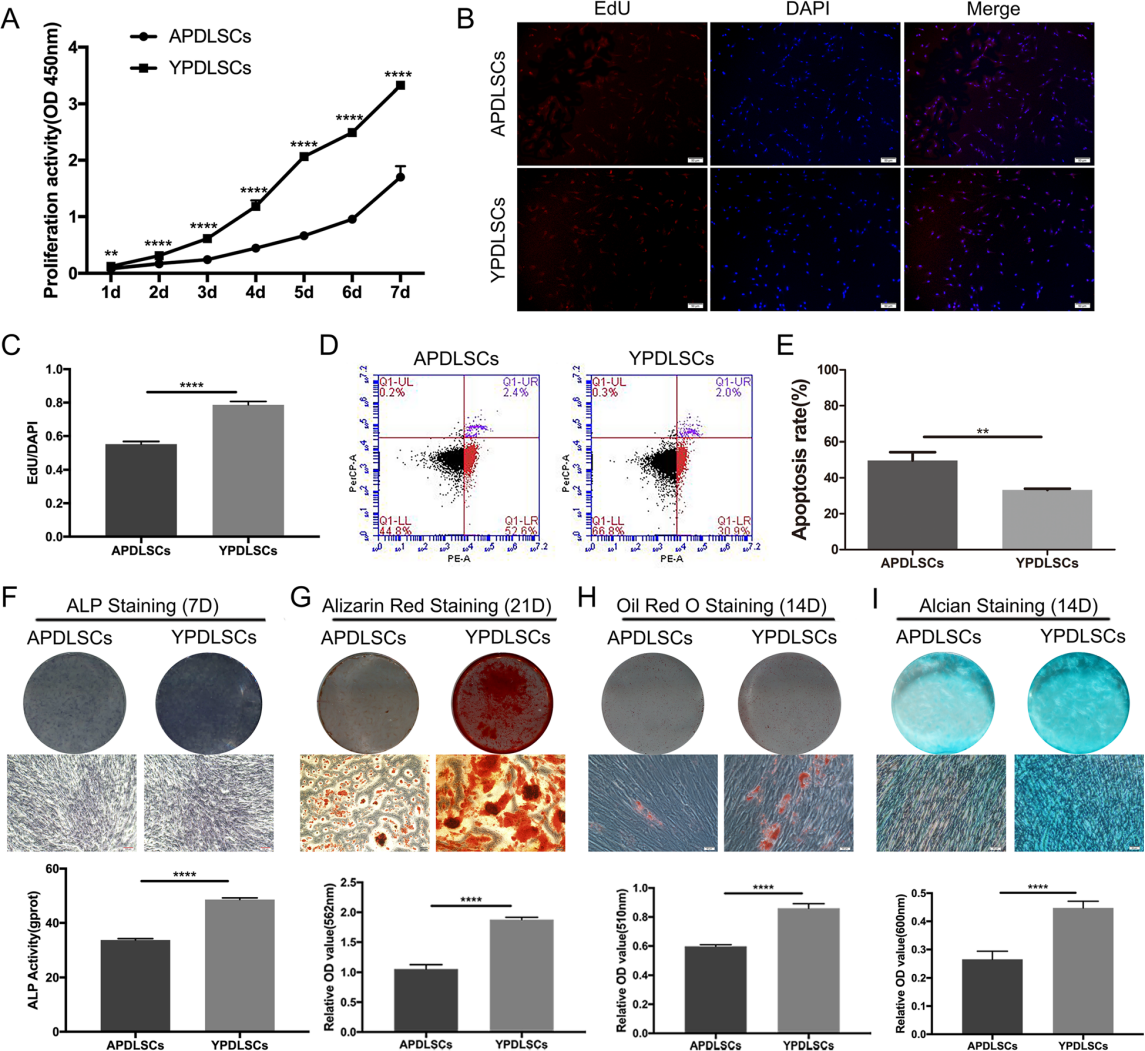
Proliferation, apoptosis, and osteogenic/adipogenic differentiation capacity of PDLSCs from different age groups. **a** CCK-8 assays showed the proliferation activity of the APDLSCs was lower than that of the YPDLSCs over 7 days of cell growth. **b**, **c** EdU staining showed that the EdU/DAPI ratio of the APDLSCs was significantly lower than that of the YPDLSCs. **d**, **e** Apoptosis assay showed that the apoptotic rate of the APDLSCs was higher than that of the YPDLSCs. **f** Both the ALP staining and the ALP activity showed that the osteogenic differentiation ability of the APDLSCs was weaker than that of the YPDLSCs. **g** Alizarin Red staining and data analysis of the ratio of dye absorption measurements for Alizarin Red in PDLSCs showed that the osteogenic differentiation ability of the APDLSCs was weaker than that of the YPDLSCs. **h** Oil Red O staining and data analysis of the ratio of dye absorption measurements for Oil Red O in PDLSCs showed that the adipogenic differentiation ability of the APDLSCs was weaker than that of the YPDLSCs. **i** Alcian Blue staining and data analysis of the ratio of dye absorption measurements for Alcian Blue in PDLSCs showed that the chondrogenic differentiation ability of the APDLSCs was weaker than that of the YPDLSCs. The young group had three independent donors, and the adult group had three independent donors. Data are presented as mean ± SD of three independent experiments (**p < 0.01, ****p < 0.0001)

The growth curves were drawn according to the results of the CCK-8 assay of cell proliferation for 7 days, and the activity of cell DNA replication activity was measured by EdU analysis. In terms of CCK-8 assays, the APDLSCs showed lower proliferative rates than YPDLSCs over 7 days of cell growth (Fig. 2a). In addition, a lower ratio of EdU-positive cells was observed in APDLSCs than in YPDLSCs (Fig. 2b, c), which suggested that the proliferation ability of APDLSCs is worse than that in YPDLSCs. Next, we detected apoptosis in the two groups by flow cytometry, which showed that the apoptotic rates of APDLSCs were higher than YPDLSCs (Fig. 2d, e). All the above results implied that age influences the number of PDLSCs through proliferation and apoptosis in vitro.

### Osteogenic/adipogenic/chondrogenic differentiation of PDLSCs from different age groups

To verify the osteogenic, adipogenic, and chondrogenic ability of PDLSCs from different age groups, we detected their ALP activity, mineralized nodules, lipid droplets, and proteoglycans. ALP staining of APDLSCs was lighter than YPDLSCs, and ALP activity was observed similar tendency (Fig. 2f). After 21 days of osteogenic induction, Alizarin Red staining and quantitative analysis demonstrated that APDLSCs accumulated fewer mineralized nodules than YPDLSCs (Fig. 2g). After 14 days of adipogenic induction, Oil Red O staining and quantitative analysis demonstrated that APDLSCs formed less lipid droplets than YPDLSCs (Fig. 2h). After 14 days of chondrogenic induction, Alcian Blue staining and quantitative analysis demonstrated that APDLSCs formed less proteoglycans than YPDLSCs (Fig. 2i). These results proved that the osteogenic, adipogenic, and chondrogenic differentiation potential of PDLSCs in the adult group is lower than that in the young group.

### PBMCs proliferation and apoptosis from different age groups

**Fig. 3:**
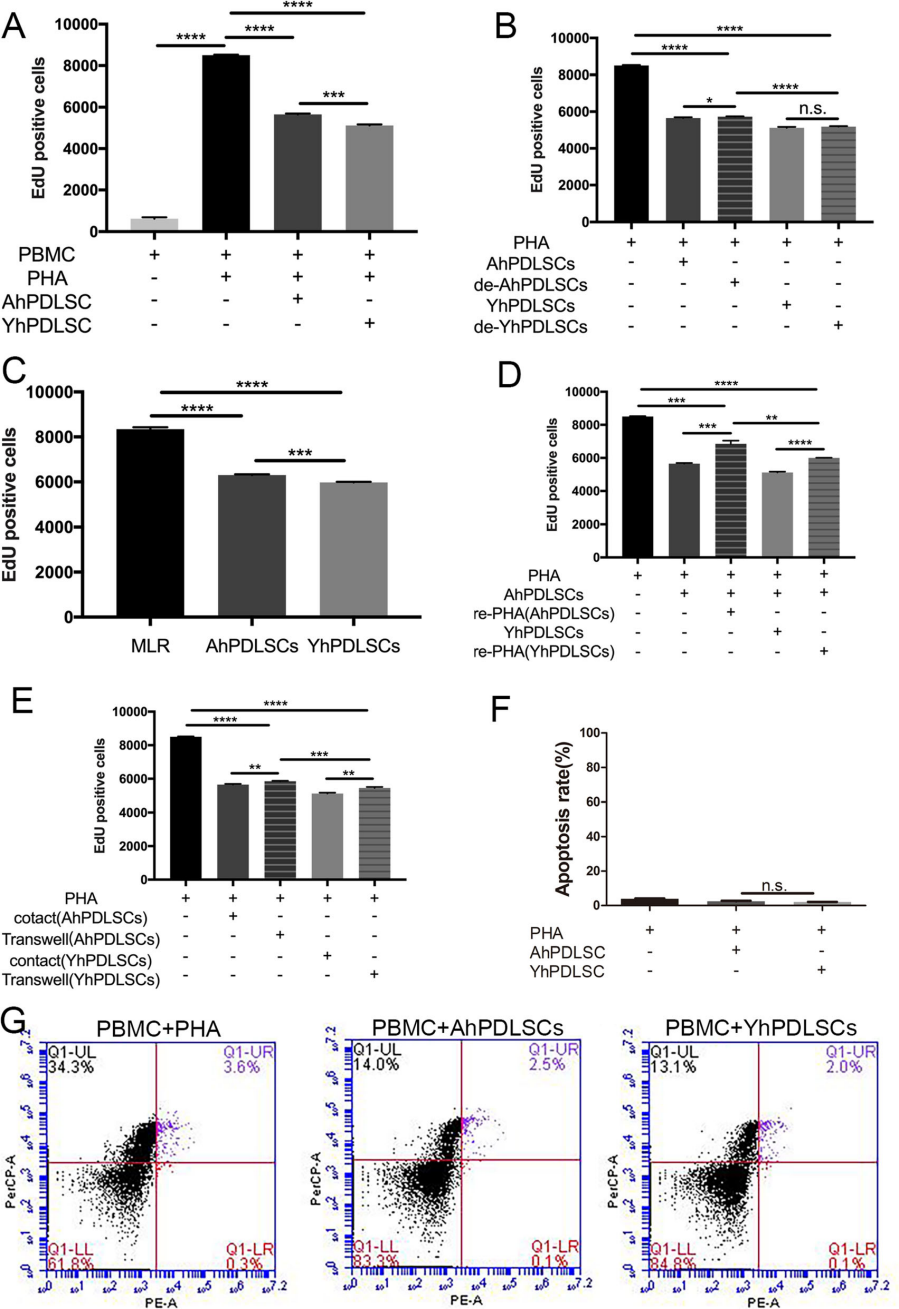
PBMCs proliferation and apoptosis. **a** APDLSCs and YPDLSCs could both significantly inhibit the proliferation of PBMCs that were stimulated by PHA, but the ability of APDLSCs to inhibit PBMC proliferation was weaker than YPDLSCs. **b** Delayed addition of PDLSCs from different age groups could also inhibit PBMCs proliferation, but the ability of APDLSCs to inhibit PBMCs proliferation was weaker than YPDLSCs. **c** On the two-way mixed lymphocyte reaction (MLR), APDLSCs and YPDLSCs could both inhibit PBMCs stimulated by allogeneic PBMCs, but the ability of APDLSCs to inhibit PBMC proliferation was weaker than YPDLSCs. **d** PBMCs inhibited by PDLSCs from different age groups could be reactivated when stimulated with PHA, but PBMCs inhibited by APDLSCs were more easily reactivated and proliferated faster than those inhibited by YPDLSCs. **e** PDLSCs from different age groups could inhibit the proliferation of PBMCs in Transwell culture, but the ability of APDLSCs was weaker than YPDLSCs. **f**, **g** There is no statistical difference in the number of apoptotic PBMCs between the co-culture group containing APDLSCs and PBMCs and the co-culture group containing YPDLSCs and PBMCs. The young group had three independent donors, and the adult group had three independent donors. Data are presented as mean ± SD of three independent experiments (*p < 0.05, **p < 0.01, ***p < 0.001, ****p < 0.0001, N.S., no significance)

To investigate the effect of PDLSCs from different age groups on allogeneic PBMC proliferation activated by mitogen, we co-cultured PDLSCs from different age groups with allogeneic PHA-stimulated PBMCs. The experimental data showed that when PDLSCs from different age groups were co-cultured respectively with PBMCs in the presence of PHA, the APDLSCs and YPDLSCs could both significantly inhibit the proliferation of PBMCs that were stimulated by PHA, but the ability of APDLSCs to inhibit PBMC proliferation was weaker than YPDLSCs (Fig. 3a). It was noticeable that delayed addition of PDLSCs from different age groups could also inhibit PBMCs proliferation, but the ability of APDLSCs to inhibit PBMC proliferation was weaker than YPDLSCs. The inhibition of PBMC proliferation by delayed addition of APDLSCs was weaker than that by the direct addition of APDLSCs, but there was no significant difference between delayed addition of YPDLSCs and direct addition of YPDLSCs (Fig. 3b). When two-way MLR was performed with co-cultures of PDLSCs collected from a third party in different age groups and PBMCs derived from two individuals, we found that the APDLSCs and YPDLSCs could both inhibit PBMCS stimulated by allogeneic PBMCS, but the ability of APDLSCs to inhibit PBMC proliferation was weaker than YPDLSCs (Fig. 3c). At the same time, we found that PBMCs inhibited by PDLSCs from different age groups respectively could be both reactivated when stimulated with PHA, but PBMCs inhibited by APDLSCs were more easily reactivated and proliferated faster than those inhibited by YPDLSCs (Fig. 3d). We also found that PDLSCs from different age groups could inhibit the proliferation of PBMCs in Transwell culture, but the ability of APDLSCs was weaker than YPDLSCs. In addition, the ability of PDLSCs to inhibit PBMC proliferation was stronger in cell-cell contact culture than that in Transwell culture (Fig. 3e), suggesting that the difference between APDLSCs and YPDLSCs in terms of proliferation of PBMCs not only was dependent on cell-cell contact but also involved soluble factors. In addition, no statistical difference in the number of apoptotic PBMCs between the co-culture group containing APDLSCs and PBMCs and the co-culture group containing YPDLSCs and PBMCs was detected (Fig. 3f, g), which implied that PBMC death was not the major cause for the observed difference in immunosuppressive ability of PDLSCs. Taken together, these results indicate that both APDLSCs and YPDLSCs suppress the proliferation of PBMCs activated by allogeneic PBMCs or stimulated by mitogen, and APDLSCs have weaker immunosuppressive ability than YPDLSCs.

### Differentially expressed genes related to differentiation and immunosuppression in PDLSCs from different age groups

**Fig. 4:**
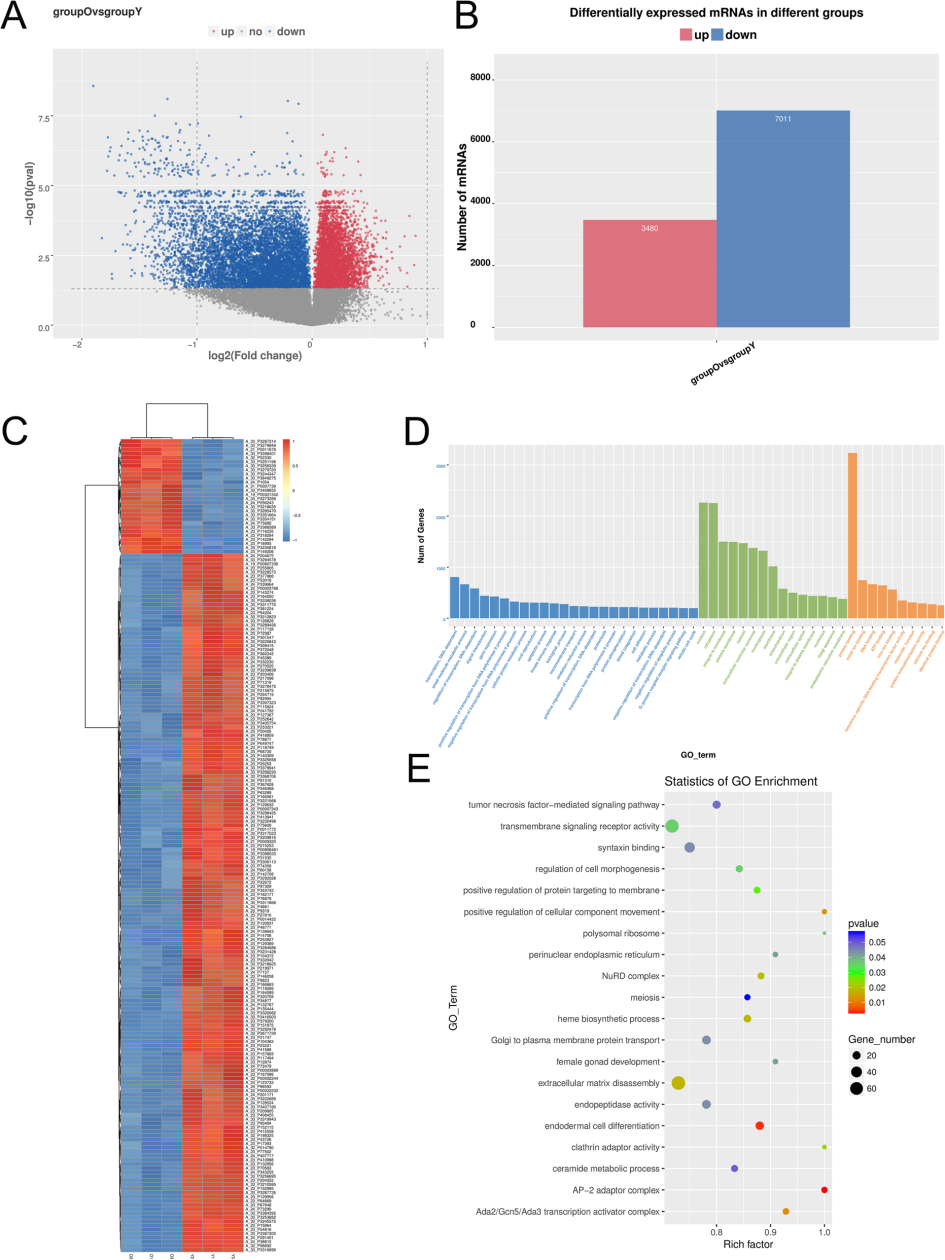
Microarray results revealed the mRNA expression profile of PDLSCs from different age groups. **a** Mapping volcanoes of PDLSCs from different age groups showed the overall distribution of differentially expressed genes. **b** Statistical analysis showed that 10,491 genes are differentially expressed, which include 3480 upregulated genes and 7011 downregulated genes. **c** Cluster analysis of PDLSCs in different age groups revealed differentially expressed genes. **d** Bar chart showed the number of differentially expressed genes based on GO term enrichment based on the functional categories of biological process, cellular component, and molecular function. **e** Scatter plot showed the statistical data of GO enrichment

**Fig. 5:**
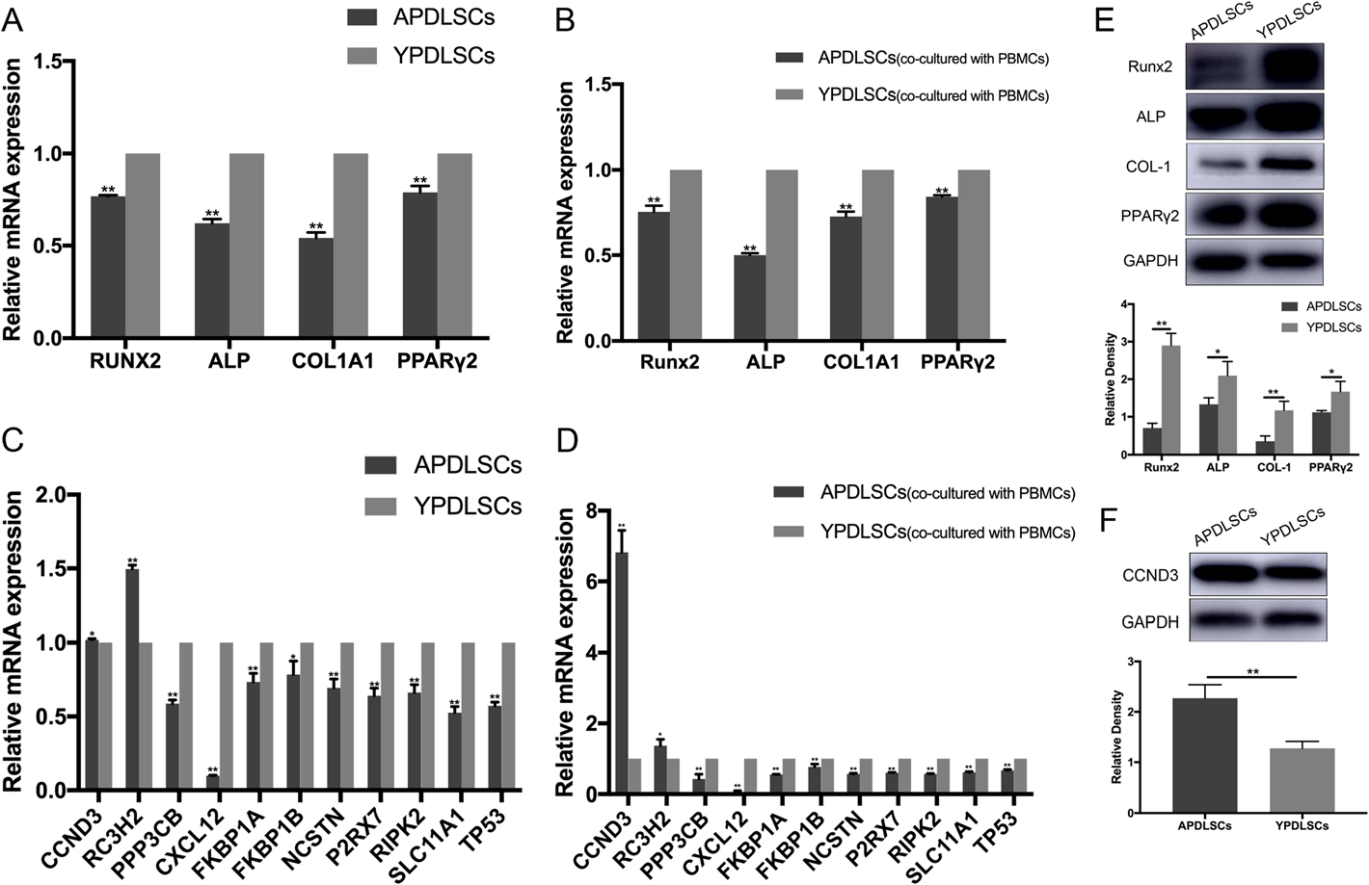
Age-related changes in the expression of genes and proteins of PDLSCs from different age groups. **a**, **c** qRT-PCR analysis showed that compared with YPDLSCs, CCND3 and RC3H2 mRNA expression in APDLSCs were upregulated, whereas Runx2, ALP, COL1A1, PPARγ2, PPP3CB, CXCL12, FKBP1A, FKBP1B, NCSTN, P2RX7, RIPK2, SLC11A1, and TP53 mRNA expression levels were downregulated. **b**, **d** qRT-PCR analysis showed that compared with YPDLSCs (co-cultured with PBMCs), the expression of CCND3 and RC3H2 in APDLSCs (co-cultured with PBMCs) increased, whereas the expression of Runx2, ALP, COL1A1, PPARγ2, PPP3CB, CXCL12, FKBP1A, FKBP1B, NCSTN, P2RX7, RIPK2, SLC11A1, and TP53 decreased. **e**, **f** Western blot analysis showed that the protein expression levels of Runx2, ALP, COL1A1, and PPARγ2 in APDLSCs were lower than YPDLSCs, whereas those of CCND3 in APDLSCs was higher than YPDLSCs. The young group had three independent donors, and the adult group had three independent donors. Data are presented as mean ± SD of three independent experiments (*p < 0.05, **p < 0.01)

Next, we used gene chip technology to analyze and identify the differentially expressed genes that were related to differentiation and immunosuppression between APDLSCs and YPDLSCs (Additional files 2 and 3). The overall distribution of differentially expressed genes was depicted as volcano plots (Fig. 4a). Statistical analysis showed that compared with YPDLSCs, a total of 10,491 differentially expressed genes were identified in the APDLSCs group, including 3480 upregulated genes and 7011 downregulated genes (Fig. 4b). At the same time, we also conducted a cluster analysis of genes to visually demonstrate the differential expression of genes in APDLSCs and YPDLSCs (Fig. 4c). Gene Ontology (GO) is a gene functional classification system that comprehensively describes the attributes of genes and gene products in organisms. We detected the distribution of the number of differentially expressed genes on GO terms enriched by biological process, cellular component, and molecular function by the bar chart (Fig. 4d). Moreover, the scatter plot also showed the statistical data of GO enrichment (Fig. 4e). Based on the above statistical analysis, we screened four differentially expressed genes that were related to the osteogenic and adipogenic ability of PDLSCs, including *Runx2*, *ALP*, *COL1A1*, and *PPARγ2*, and found 14 differentially expressed genes associated with immunosuppressive ability of PDLSCs, including *CCND3*, *RC3H2*, *PPP3CB*, *CXCL12*, *FKBP1A*, *FKBP1B*, *NCSTN*, *P2RX7*, *RIPK2*, *SLC11A1*, *TP53*, *TNFSF14*, *RC3H1*, and *TNFRSF4*. Then, to verify the reliability of microarray results, 18 differentially expressed genes were quantitatively analyzed by qRT-PCR. The qRT-PCR results showed that compared with YPDLSCs, the expression levels of *CCND3* and *RC3H2* in APDLSCs were upregulated, whereas the expression levels of *Runx2*, *ALP*, *COL1A1*, *PPARγ2*, *PPP3CB*, *CXCL12*, *FKBP1A*, *FKBP1B*, *NCSTN*, *P2RX7*, *RIPK2*, *SLC11A1*, and *TP53* were downregulated (Fig. 5a, c), which coincided with the findings of microarray assay. However, the results of *RC3H1*, *TNFRSF4*, and *TNFSF14* were discordant to those of microarray assay (Additional file 4: Fig. S2). Subsequently, we verified the expression of these genes in the YPDLSCs and APDLSCs co-cultured with PBMCs, and the trends were similar to those of non-co-cultured PDLSCs (Fig. 5b, d). Meanwhile, compared with YPDLSCs, the protein expression levels of Runx2, ALP, COL1A1, and PPARγ2 in APDLSCs decreased (Fig. 5e), whereas those of CCND3 in APDLSCs increased (Fig. 5f). Altogether, these results suggest that Runx2, ALP, COL1A1, and PPARγ2 are involved in regulating the osteogenic/adipogenic differentiation of PDLSCs from different age groups, and CCND3 is involved in regulating the immunomodulation of PDLSCs from different age groups.

## Discussion

According to previous studies, we know that PDLSCs have the capability of self-renewal and can differentiate into various tissue-specific cells, such as osteoblasts, chondrocytes, and adipocytes [25, 26]. In recent years, based on the expanding understanding of the regeneration and potential mechanism of PDLSCs, we further found that PDLSCs expressed low immunogenicity and had immunoregulatory capability, and thus, transplantation of allogeneic PDLSCs into the host would not result in immune rejection [18]. Meanwhile, we have known that age-related loss of beneficial function of PDLSCs may be related to their pluripotency in periodontal tissue engineering [27]. Therefore, this study compared and analyzed the age-related changes in the biological and immunological features of PDLSCs and their possible mechanisms.

Previous studies [8, 13] have shown that PDLSCs could be divided into two groups, namely, YPDLSCs and APDLSCs, based on a certain comparative age range. Our data showed that the positive expression rates of STRO-1 and CD146 in PDLSCs decreased with age, which was consistent with the findings of previous studies [13]. Earlier investigations have shown that the surface-specific markers of MSCs, namely, STRO-1 and CD146, can reflect changes in the number of PDLSCs [12]. Moreover, STRO-1^+^ cells in MSCs have significantly enhanced inhibitory effects on lymphocyte proliferation compared with STRO-1^−^ cells, i.e., STRO-1^+^ cells impart stronger immunoregulatory effects than STRO-1^−^ cells, which supports our observations [28, 29]. This suggests that the number and immunoregulatory characteristics of PDLSCs decrease with age, which coincides with the subsequent results of this study. Then, we found that with increasing donor age, the proliferation rate of PDLSCs decreased, whereas the level of apoptosis increased. These results agree with the findings of previous studies [27], indicating that there is a negative correlation between aging and the proliferation activity of PDLSCs. Active proliferation means that YPDLSCs have a greater self-renewal ability than APDLSCs. In addition to the ability of proliferation, we found that compared with YPDLSCs, the osteogenic, adipogenic, and chondrogenic differentiation potential of APDLSCs was significantly reduced, i.e., PDLSCs with greater osteogenic/adipogenic/chondrogenic differentiation potential can be obtained within a short period of time when these are derived from young individuals. Some conflicting reports have been published. For example, Baker et al. [10] found that the adipogenic potential of BMSCs increased with age, whereas Choudhery et al. [30] reported no change in adipogenic potential of adipose tissue-derived mesenchymal stem cells (AT-MSCs) from individuals of advanced age. These discrepancies may be due to variations in gene expression of different types of MSCs. Taken together, our study demonstrates that aging is associated with the deterioration of biological characteristics of PDLSCs.

In accordance with previous reports [20], YPDLSCs expressed HLA-І but not of HLA-II DR, CD80, and CD86. However, the present study did not observe any statistically significant difference between APDLSCs and YPDLSCs. As the secondary signaling molecules of T cell activation, these proteins are co-stimulatory molecules that play an important role in host immune responses and immunoregulation [31, 32]. Therefore, the results of the present study show that both YPDLSCs and APDLSCs express low immunogenicity, i.e., donor age does not affect the immunogenicity of PDLSCs. To test the immunosuppressive functions of PDLSCs, we compared the effects of PDLSCs in different age groups using lymphocyte proliferation and mixed lymphocyte response (MLR) experiments. We conclude that PDLSCs from individuals of different age groups impart strong immunosuppressive effects on the proliferation of allogeneic PBMCs, whether in cell-cell contact culture or Transwell cultures, whereas the immunosuppressive effect of PDLSCs from adult individuals was weaker than that of PDLSCs from young subjects. These findings agree with the results of previous reports using other kinds of MSCs, i.e., donor age influences immunoregulation in MSCs [14, 33, 34], although MSC inhibition of T cell proliferation is preserved in the elderly [35]. Furthermore, we found that the difference in immunosuppressive ability between APDLSCs and YPDLSCs is not related to the apoptosis of PBMCs. These findings are similar to those of previous studies that observed that the anti-proliferative activity of MSCs is due to cell cycle arrest of active T cells at the G0/G1 phase and not by apoptosis [36]. Therefore, our study demonstrates that aging can degrade the immunological characteristics of PDLSCs.

Previous studies have found that BMSCs from donors of different ages have significant differences in gene expression levels [37, 38]. Hence, to explore the mechanisms that cause differences in the biological and immunological characteristics of PDLSCs from young and adult individuals, we performed microarray analysis and used qRT-PCR and western blot analyses to verify our microarray results to identify genes that may play a key role in the age-related decline of PDLSCs. The results showed that the expression of genes related to osteogenic/adipogenic and immunosuppressive capacity in PDLSCs was regulated to varying degrees with increasing age. The expression of osteoblastic markers Runx2, ALP, and COL1A1 revealed age-related decline in both gene and protein expression levels, which supported the observed decline in osteogenic differentiation ability of PDLSCs due to aging. In accordance with previous reports, age is associated with changes in the differentiation ability of BMSCs, i.e., they become less bone and more fat, which is driven by the increase in the expression of PPARγ2, a specific transcription factor of adipocytes [39–41]. However, in this study, the expression of adipogenic marker PPARγ2 showed age-related decline in both gene and protein expression levels. This may explain the difference in the effect of aging on the adipogenic differentiation between PDLSCs and BMSCs. Furthermore, in terms of immunosuppressive capacity, whether PDLSCs not co-cultured with PBMCs or PDLSCs co-cultured with PBMCs, compared with YPDLSCs, *CCND3*, and *RC3H2*, were upregulated in APDLSCs, whereas *CXCL12*, *FKBP1A*, *FKBP1B*, *NCSTN*, *P2RX7*, *PPP3CB*, *RIPK2*, *SLC11A1*, and *TP53* were downregulated in APDLSCs. Among these differentially expressed genes, we found that compared with non-co-cultured PDLSCs, changes in the expression of CCND3 were the most significant in PDLSCs co-cultured with PBMSCs. Therefore, we again assessed CCND3 protein expression levels, which coincided with the gene expression levels. As indicated previously, CCND3 is known to be involved in regulating the cell cycle of activated T lymphocytes [42], and cyclin D3−/− animals cannot normally expand immature T lymphocytes [43]. Furthermore, in addition to its role in cell cycle progression, cyclin D3 imparts significant effects on PPARγ activity and subsequently on adipogenesis [44, 45]. Hence, the gene expression patterns described in the current study may provide novel markers for aging in PDLSCs. In the future, we will further explore and clarify the mechanism underlying these differentially expressed genes.

Our comprehensive experimental study has confirmed the relationship between age and the biological and immunological characteristics of PDLSCs, thereby providing some valuable information for basic research and clinical applications. First of all, the proliferation, differentiation, and immunosuppressive ability of PDLSCs from young individuals are better than those of adult PDLSCs, which means that young PDLSCs should be selected for the treatment of periodontitis or tooth regeneration. Second, published studies have shown that cryopreservation, as a feasible option for the long-term preservation of biomaterials such as stem cells [46, 47] and embryos [48], is an important method for maintaining cell activity and function, providing a pathway for the success of regenerative medicine and tissue engineering. Moreover, our previous studies have verified that cryopreservation does not change the structural integrity and functional viability of PDLSC sheets [49], allowing us to provide cellular products when patients need these. Therefore, we should pay attention to the storage of PDLSCs from young individuals for later use when the donor needs clinical treatment at a later age or for allotransplantation in other elderly people.

## Conclusions

Our results provide novel insights into the differences in the characteristics of PDLSCs from young and adult individuals and elucidate the potential molecular mechanism of PDLSC aging. For therapeutic purposes, it is thus essential to recognize and further understand the mechanism of age-related functional degradation of PDLSCs to formulate effective strategies to reverse aging in PDLSCs and to develop schemes in PDLSC-based tissue engineering and regenerative medicine.

## Supplementary information

**Additional file 1: Figure S1.** Both APDLSCs and YPDLSCs expressed CD105 positively. Data are presented as mean ± SD of triplicates of six independent experiments (N.S., no significance).

**Additional file 2.** mRNA_differential_expression/groupAvsgroupY_Diff_Analysis.xlsx.

**Additional file 3.** mRNA_differential_expression/groupAvsgroupY_GO_Enrichment.xlsx.

**Additional file 4: Figure S2.** Age-related changes in the expression of genes of PDLSCs from different age groups. qRT-PCR results showed that compared with YPDLSCs, RC3H1 and TNFSF4 mRNA expression in APDLSCs were up-regulated, while TNFSF14 mRNA expression were down-regulated. Those results were not consistent with those of microarray. Data are presented as mean ± SD of triplicates of six independent experiments (**p < 0.01).

## Data Availability

All data and materials associated with this study are present in this published article.

## Abbreviations

PDLSCs: Periodontal ligament stem cells
MSCs: Mesenchymal stem cells
PBMCs: Peripheral blood mononuclear cells
BMSCs: Bone marrow mesenchymal stem cells
AT-MSCs: Adipose tissue-derived mesenchymal stem cells
PBS: Phosphate-buffered saline
α-MEM: Alpha-modified Eagle’s medium
RPMI-1640: Roswell Park Memorial Institute-1640
FBS: Fetal bovine serum
MLR: Mixed lymphocyte response
CCK-8: Cell Counting Kit-8
EdU: 5-Ethynyl-2′-deoxyuridine
ALP: Alkaline phosphatase
qRT-PCR: Quantitative reverse transcriptase-polymerase chain reaction
