# Supplementary figures and images for: The effect of aging on the biological and immunological characteristics of periodontal ligament stem cells
Source: Stem Cell Res Ther. 2020 Jul 29;11:326. doi: 10.1186/s13287-020-01846-w (PMC7392710; doi:10.1186/s13287-020-01846-w)

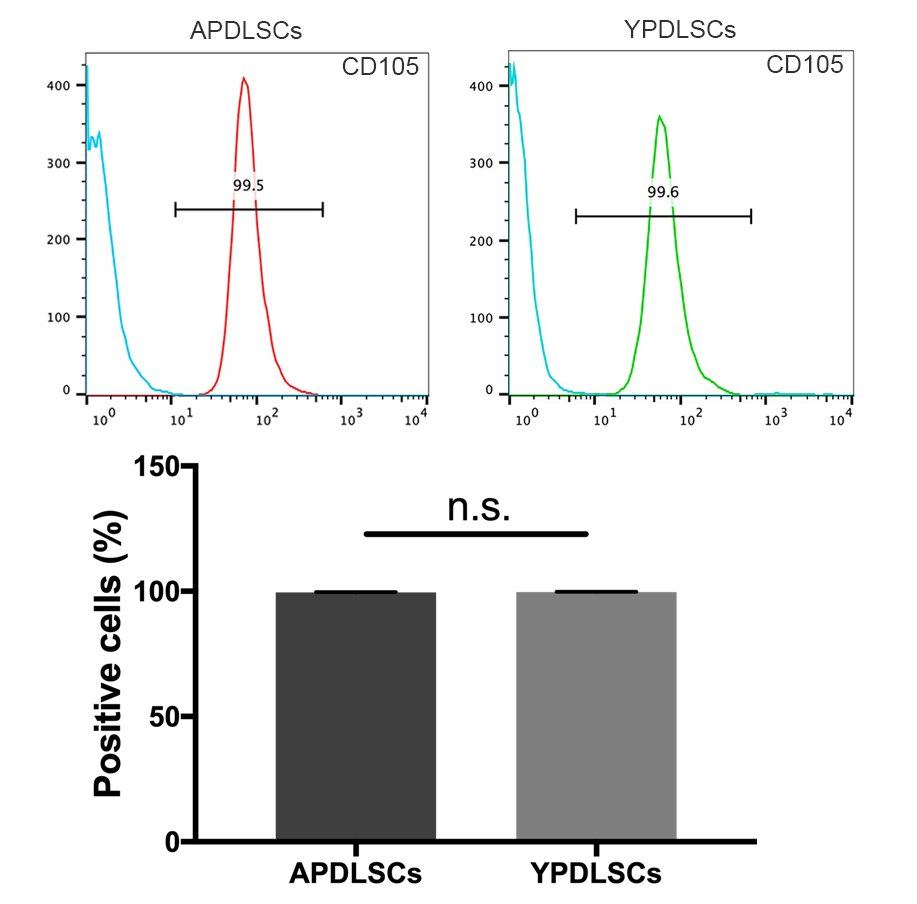

Supplement: Supplementary file 1 — Additional file 1: Figure S1. Both APDLSCs and YPDLSCs expressed CD105 positively. Data are presented as mean ± SD of triplicates of six independent experiments (N.S., no significance). [file 13287_2020_1846_MOESM1_ESM.tif]

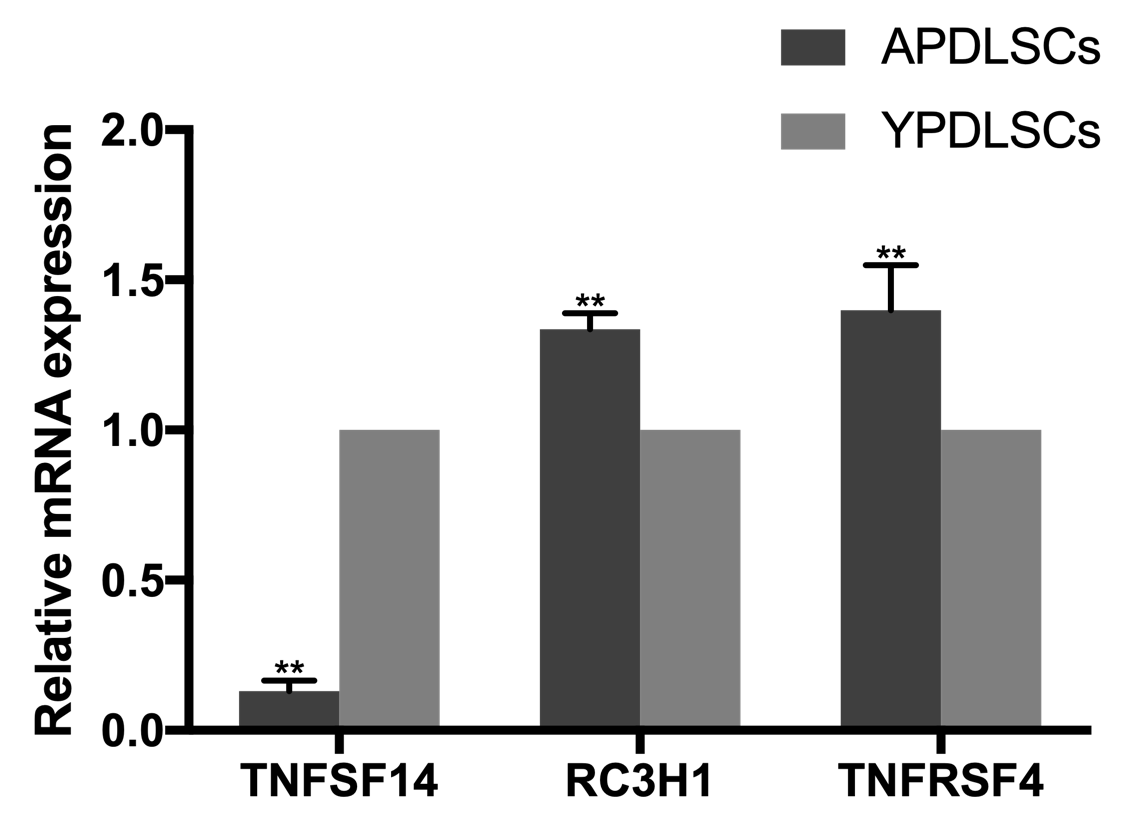

Supplement: Supplementary file 4 — Additional file 4: Figure S2. Age-related changes in the expression of genes of PDLSCs from different age groups. qRT-PCR results showed that compared with YPDLSCs, RC3H1 and TNFSF4 mRNA expression in APDLSCs were up-regulated, while TNFSF14 mRNA expression were down-regulated. Those results were not consistent with those of microarray. Data are presented as mean ± SD of triplicates of six independent experiments (**p < 0.01). [file 13287_2020_1846_MOESM4_ESM.tif]
